# The incidence, presenting clinical findings and treatment patterns of Birdshot Retinochoroiditis in a high-prevalence region: findings from Northern Ireland, England and Wales

**DOI:** 10.1038/s41433-023-02425-y

**Published:** 2023-02-10

**Authors:** Rana Khalil, Harry Petrushkin, Angela Rees, Mark Westcott

**Affiliations:** 1 Royal College of Surgeons in Ireland, 123 St Stephen’s Green, Dublin, D02 YN77 Republic of Ireland; 2 Moorfields Eye Hospital NHS Foundation Trust, 162 City Road, London, EC1V 2PD United Kingdom; 3 Great Ormond Street Hospital for Children, Great Ormond Street, London, WC1N 3JH United Kingdom; 4 UCL Institute of Ophthalmology, 11-43 Bath St, London, EC1V 9EL United Kingdom; 5 Queen Mary University of London, Mile End Rd, Bethnal Green, London, E1 4NS United Kingdom

**Keywords:** Epidemiology, Uveal diseases

## Abstract

**Background:**

Birdshot Retinochoroiditis (BSRC) is a rare, chronic posterior uveitis that is strongly associated with HLA-A*29.2 positivity. To date, no robust incidence studies of BSRC have been undertaken. We present the first epidemiological study of BSRC in a high-prevalence region.

**Methods:**

In collaboration with the British Ophthalmological Surveillance Unit, all new cases of BSRC between May 2017 and June 2019 were prospectively collected. Presenting demographics, symptoms, signs and treatment modalities were collected. A follow-up questionnaire twelve months later was also sent.

**Results:**

Thirty-seven confirmed cases meeting the reporting criteria were identified. Twenty-three cases had both baseline and follow-up data. The total population incidence of BSRC was 0.035 cases per 100,000 person-years [95% CI 0.025–0.048 cases per 100 000 people]. 97.3% were HLA-A*29 positive. The median age was 46 years, with females making up 78% of patients. There were no significant differences in the latitudinal incidence of BSRC. At presentation, floaters were the most common symptom. Optic disc swelling was the most common sign. Mean presenting visual acuity was independent of symptom duration. Combined systemic corticosteroids and immunomodulatory therapy were the most common treatments at baseline and follow-up. Intravitreal steroids were equally popular at follow-up.

**Conclusions:**

This study provides the first nationwide estimate of the incidence of BSRC in a high-prevalence region. Cases were more common in females, with a broad range of presentation ages. No significant latitudinal effect of incidence was identified. Systemic therapy with steroids and IMT remain the most common treatments.

## Introduction

Birdshot retinochoroiditis (BSRC) is a rare inflammatory disease of the choroid with an unknown aetiology. BSRC is characterised by a progressive, bilateral, chronic posterior uveitis with a distinctive clinical phenotype. BSRC is named after the characteristic appearance of choroidal lesions that have the appearance of a shotgun splatter. It has the strongest known association to Human Leukocyte Antigen of any disease, particularly HLA-A*29.2, and largely affects middle-aged white individuals of Northern European descent [1]. The mean age of disease onset is 53 years old [2], with a slight female preponderance [1].

An estimate of the true incidence of BSCR, or indeed any rare disease poses a challenge due to case ascertainment. Current data reveals that BSRC may make up to 1.5% of all uveitis cases seen in specialist uveitis clinics, however, the true prevalence of BSCR is certainly not known, and can best be estimated by obtaining information from tertiary referral centres which does not represent a true population prevalence [1, 3, 4]. Indeed, the total population denominator that is subserved by single tertiary centres is largely unknown, which places large confidence limits on any calculation of the incidence.

Without timely and appropriate treatment, BSRC can result in permanent progressive visual decline, with associated retinal and choroidal atrophy [5]. We present a two-year analysis of incidence, presenting features and treatment trends in BSCR in Northern Ireland, England and Wales. To the authors’ knowledge, this is the first prospective surveillance study designed to estimate the incidence of BSCR in this high-prevalence region.

## Materials/Subjects and methods

Cases from England, Wales and Northern Ireland were prospectively identified using the British Ophthalmological Surveillance Unit (BOSU) monthly reporting card system [6]. BOSU is an active surveillance system that was established in 1997 for the reporting of rare eye diseases in the UK and Ireland. It allows for the identification and accurate epidemiological evaluation of relevant ocular conditions by consultant ophthalmologists. Several trials comparing the outcomes of active and passive surveillance systems found the active arm to report at least twice as many cases as the passive arm [7–9]. Each month, all ophthalmologists on the specialist register are sent a yellow reporting card with a list of current conditions under surveillance. Once reported, the appropriate study investigators are notified and a questionnaire is sent to the reportee for further collection of clinical, diagnostic and demographic data for study analysis [10].

For the twenty-four-month study period between May 2017 and June 2019 inclusive, ophthalmologists were asked to notify the study investigators, through the BOSU, of any newly presenting patients with BSRC. A total of twenty-six ophthalmologists engaged with this study, of whom fourteen are uveitis specialists.

At the time of study design, we chose to use the Levinson criteria for the diagnosis of new BSRC cases [11]. There should have been no other evident cause of uveitis, including inflammatory or infectious uveitis, and neoplastic ocular masquerade syndromes. Supportive criteria included HLA-A*29 haplotype positivity, and all patients were required to undergo HLA-A*29 haplotype testing. However, as per the Levinson criteria, we elected not to restrict our definition to include only HLA-A*29 haplotype-positive cases [11].

Reporting ophthalmologists were sent a questionnaire at initial diagnosis and twelve months. Ophthalmologists who did not return either questionnaire were sent at least two reminder letters to increase the response rate. Duplicate reports were excluded using a combination of first half of the post-code and date of birth.

We split the study region into three main zones, divided broadly by latitude, using data from the nine enterprise zones of the Office for National Statistics [12]. For the purposes of this study, we considered Northern Ireland to be part of the ‘upper zone’. The reason for this was to investigate any relationship between increasing latitudes, as anecdotal evidence suggests that even within Europe, BSCR is much more prevalent in the north compared to the south.

For the purposes of calculating incidence rates of exclusively White British populations at risk for BSRC, we obtained information on estimated population and ethnic classification by region from the latest accessible Census Day results of 27 March 2011 [13]. We only included the estimates of ‘White English/ Welsh/ Scottish/ Northern Irish/ British’ for England/Wales [14, 15] and ‘White’ for Northern Ireland [16] in this study. We obtained information on population estimates and ethnicity from the Office for National Statistics based on 2011 census data.

Data was recorded in a Microsoft Access database. Best corrected visual acuity (BCVA) was converted to Logarithm of the Minimum Angle of Resolution (LogMAR) and treated as a continuous variable [17]. Between-eye LogMAR acuities were found to be highly correlated and therefore mean of left and right-eye LogMAR acuities were calculated to give a global score for each patient.

## Results

Seventy-seven cases were reported to the BOSU during the twenty-four-month study period. Thirteen were duplicate reports, while twenty cases were incorrectly reported before the study period and so excluded. Of the remaining forty-four cases, six had incomplete data and one was lost to follow-up. This yielded a total of thirty-seven eligible cases, with a response rate of 84%. Of these, only twenty-three responded to the follow-up questionnaire.

The total population incidence of BSRC was found to be 0.035 cases per 100,000 person-years [95% CI 0.025–0.048 cases per 100,000 people] [18].

### Patient characteristics

**Fig. 1:**
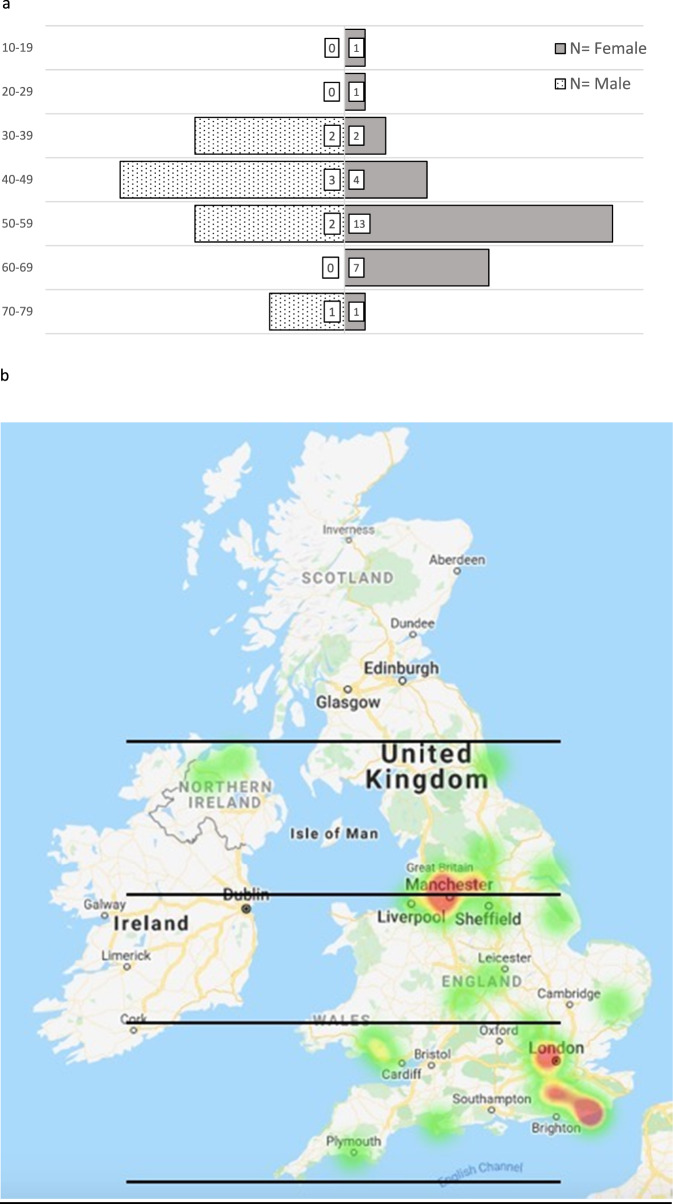

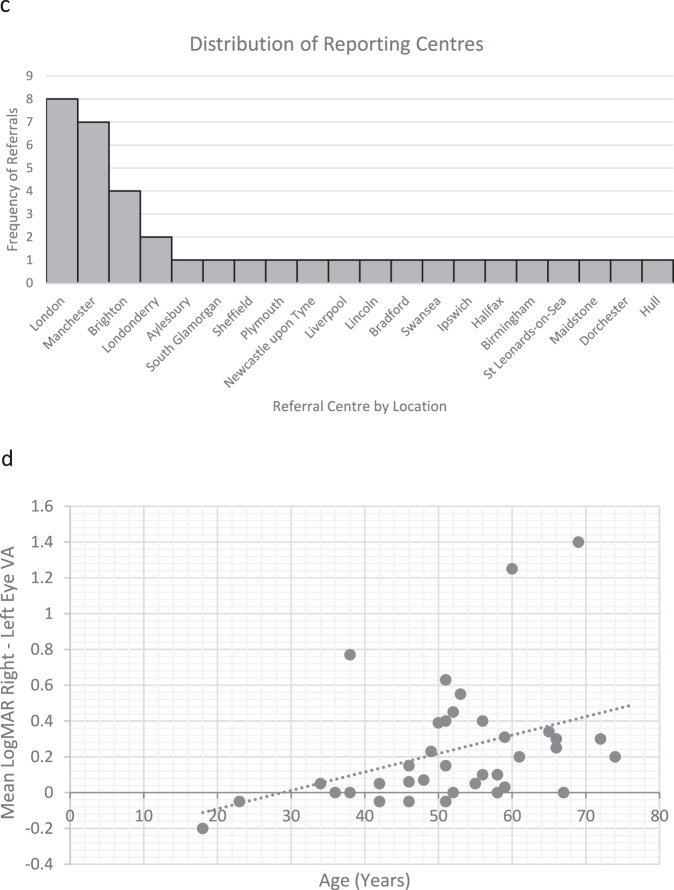

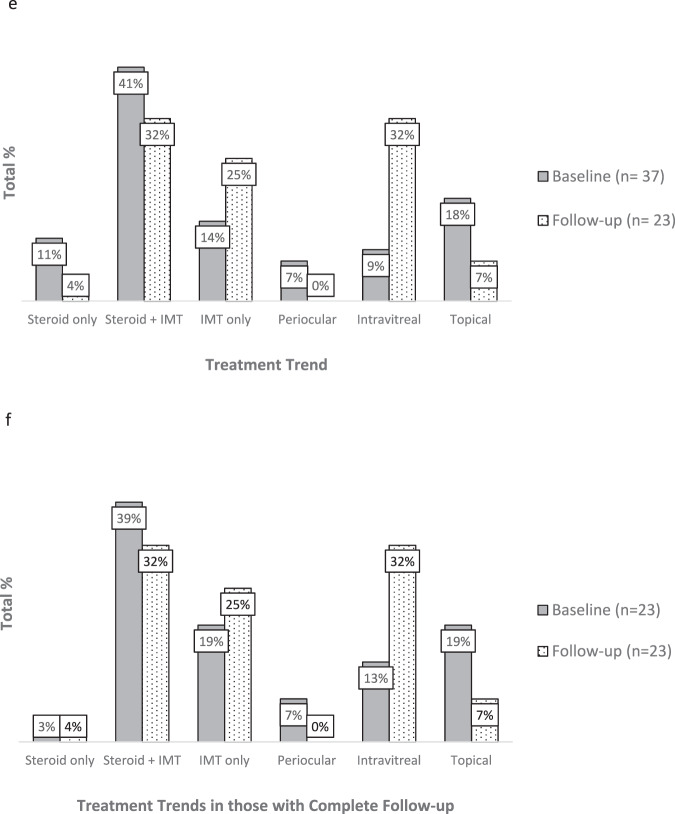
General characteristics and geographic distribution. **a** Age and Gender Distribution. **b** Heat Map by Patient Post-code. **c** Distribution of Reporting Centres. **d** Mean Right–Left Presenting Acuities versus Age. **e** Treatment trend in all patients over 12 months. **f** Treatment trend in those with complete data over 12 months.

97.3% of cases were HLA-A*29 positive. The median age was forty-six years (range 18–74), with females making up 78% of patients (Fig. 1a). 100% of recorded cases were born in the UK. Of the thirteen ethnicities recorded, all were Caucasian. Unfortunately, ethnicity was only added as an option on the form later on, therefore was not consistently asked over the duration of the study. One case (2.7%) reported a positive family history of BSRC. No one reported a prior history of rhegmatogenous retinal detachment (RRD) or psoriasis.

In the 2011 census, 46,913,135 people identified as White British, making up 81% of the population of Northern Ireland, England and Wales. We calculated incidence rates of exclusively White British populations at risk for BSRC. Although our findings suggest the incidence is highest in the most northern latitude band, this was not statistically significant (Table 1a). This is because the 95% CI of incidence was large and overlapped between the three latitude zones. However, this finding warrants further study. Figure 1b reveals the mapping of incidence rates according to patient post-codes, which is comparable to the distribution of the referral centres (Fig. 1c), suggesting that most patients were referred locally.

**Table 1:** Incidence and presenting features.

a: Incidence of BSRC in White British Population			
	Incidence per 100,000 person-year	95% Confidence Interval	Latitude bands
Upper Zone	0.047	0.019 to 0.097	55.246°–53.454° N
Middle Zone	0.027	0.006 to 0.079	53.454°–51.985° N
Lower Zone	0.031	0.017 to 0.053	51.985°–49.958° N

b: Presenting features			
Presenting symptoms	Presenting structural abnormalities	Presenting functional abnormalities	
Floaters (72.9%), *n* = 37	Optic disc swelling (24.3%), *n* = 37	Abnormal ERG (82%), *n* = 11	
Poor vision (60.8%), *n* = 37	CMO (21.6%), *n* = 37	Abnormal visual fields (47%), *n* = 17	
Photopsia (32.4%), *n* = 37	Optic disc pallor (8.1%), *n* = 37	Abnormal colour vision (11%), *n* = 18	
Nyctalopia (13.5%), *n* = 37			

c: Mean Right–Left Eye Presenting Acuities, by Gender			
Gender	*N*	Group means of presenting LogMAR acuities in both eyes	F, Significance
Female	29	0.25+/−0.36	0.233, *P* = 0.633
Male	8	0.19+/−0.28	

### Patient-reported symptoms

The median duration of symptoms until diagnosis was thirty-two weeks in females (range 4–208 weeks) and twenty-one weeks in males (range 1–156 weeks). Over half of patients in both groups had symptoms exceeding six months. The most common initial symptoms were floaters (72.9% of eyes), poor vision (60.8%), photopsia (32.4%), and nyctalopia (13.5%) (Table 1b).

### Ophthalmic findings

Mean right–left LogMAR presenting acuity worsened significantly with age (*r*_sqr_ = 0.44, *p* = 0.007); see Fig. 1d. Table 1c shows the distribution of mean right–left eye presenting acuities, according to gender. Mean acuities of female patients were slightly worse than worse than male patients, although this was not statistically significant. Qualitative review of LogMAR according to symptom duration showed no relationship. To explore further, patients were divided up into 2 groups: those with presenting symptoms <32 weeks, and >32 weeks. Mean LogMAR visual acuity (VA) did not differ significantly between groups, further supporting the finding that mean presenting VA was independent of duration of symptoms.

Less than one-third of patients were identified as having CMO in at least one eye at diagnosis. We performed a t-test focusing on CMO. At diagnosis, 10 patients had CMO in at least 1 eye and 25 patients did not (2 patients had incomplete data). Of the CMO group, 2 had other possible causes of poor vision (dense amblyopia, macular hole) and were excluded from analysis. Of the non-CMO group, a further 2 were excluded for similar reasons. Mean VA in the first group was LogMAR 0.32 (SD 0.24), whereas the second group had mean LogMAR 0.097 (SD 0.17). This was statistically significant (*p* = 0.007, equal variances assumed). No patients developed new CMO over the study period. Due to the small number of patients in this study, we were unable to consider the effects of other factors (such as cataract) on visual outcomes.

The most common clinical signs at presentation were optic disc swelling (24.3%), followed by cystoid macular oedema (21.6%) and optic disc pallor (8.1%) (Table 1b). We did not receive sufficient reporting information on colour, visual field or electrodiagnostic testing, with over half of reporting ophthalmologists indicating ‘unknown’ for at least one of these parameters. Thus, only a small sub-analysis as related to presenting visual acuity is presented (supplementary material). It can be observed that abnormal colour vision or visual fields were associated with poor visual acuity (>LogMAR 0.2), whilst normal parameters were more likely to have good presenting vision (≦LogMAR 0.2). Interestingly, this does not hold true for ERG testing, whereby an almost equal number of patients with abnormal results had vision either better or worse than LogMAR 0.2. This highlights the fact that presenting VA may be normal even in the presence of significant electrophysiological disturbance.

### Treatment trends

The six categories of treatment were systemic steroids only, systemic steroids plus immunomodulatory therapy (IMT), IMT alone, topical steroids, and local steroids comprising of peri-ocular or intravitreal therapy. While the first three modalities are mutually exclusive, the latter three are not, and it was possible for clinicians to combine various modalities such as IMT plus intravitreal. At baseline, systemic therapy was the most common form of treatment (66%), followed by topical (18%) and local (16%) therapy. A combination of steroids and IMT (41%) was the most popular systemic choice, followed by IMT alone (14%), and steroids alone (11%) (Fig. 1e). In terms of local therapy, intravitreal comprised 9% whereas periocular made up 7% of total treatments given. At twelve months, systemic therapy remained the most commonly used (61%). Of those 23 patients with matched data, there was a trend away from combined steroid and IMT therapy (39 to 32%), and towards IMT only (19 to 25%) (Fig. 1f). There was a negligible increase in the proportion of those receiving systemic steroid only (3 to 4%). For local therapies, a significant drop in periocular (7 to 0%) and topical (19 to 7%) treatments was noted over one year, with a corresponding increase in intravitreal steroids (13 to 32%).

## Discussion

It is very difficult to ascertain the true incidence rate of a rare disease as BSCR. Current data is derived from major tertiary referral centre studies, which is likely over-represented due to referral bias. In the Manchester Uveitis Clinic study, BSRC made up 1.2% of new diagnoses [19]. Similarly, Shah et al. estimated that BSRC makes up 0.6–1.5% of uveitis patients in tertiary referral centres, comprising 6–7.9% of patients with posterior uveitis [4]. In our study, the population incidence of BSRC in the White British population was 0.035 cases per 100,000 person-years [95% CI 0.025–0.048 cases per 100,000 people]. The authors do not note a significant difference between the location of reporting centres and patient post-codes, suggesting that most patients were referred locally. Not all major uveitis centres were well-engaged in the study, with Manchester and London being over-represented (see Fig. 1c). We do not have an explanation for this. Due to the rarity of this condition, poor engagement from other major centres, and the likelihood of delayed or missed diagnoses in real-life settings, it is possible that there was an under-reporting of new BSCR cases. However, this study’s extended endpoints and additional year of data acquisition aimed to reduce the number of false positives, as patients initially mis-reported will have turned out to have another disease. Thus, within the confines of the BOSU architecture, this represents the most accurate data we can currently obtain in Northern Ireland, England and Wales.

BSRC is known to be a disease of middle-aged Caucasians of Northern European descent [4, 20], with genetic studies suggesting there is an element of familial aggregation, with approximately 3–4% of patients having a positive family history [21–24]. Very few case reports exist of BSRC in patients of non-White descent [20, 25–27]. We elected to use the Levinson diagnostic criteria for consistency with previous reports of BSRC from large tertiary centre studies, whereby HLA-A*29 positivity was supportive but not required for the diagnosis [11]. Our findings support the position that a HLA-A*29 negative BSRC phenotype is extremely rare, if it exists at all, and for practical purposes excluding such patients would not materially affect our incidence calculation. Over 95% of BSCR patients are known to carry the HLA-A*29:02 allele, as opposed to approximately 7% of the general population, making it the strongest documented HLA association in any human disease [28]. Although previously thought to confer susceptibility [29], HLA-A*29:02 alone cannot explain preponderance to BSRC. One study showed that HLA-A*29:02 is present at least ten times less frequently in Asian Americans compared to White Americans, however, this is comparable between African Americans and Hispanics [30].

Interestingly, there have been several case reports of BSCR in association with either RRD or psoriasis [31–36], with one Dutch study reporting RRD rates to be particularly high in those with retinitis-associated posterior uveitis compared to the general population [32]. These observations encouraged us to examine these factors, however, in our study, no patients had a history of RRD or psoriasis.

BSRC patients commonly face delays in diagnosis, with over half of our patients presenting with at least six months of symptoms. This is because the initial retinal signs can be subtle, the symptoms non-specific, and visual acuity often remains preserved in the early stages of the disease [37]. In a large systematic review, Shah et al. found that on presentation, 76% of patients had a visual acuity of 6/12 or more in the better-seeing eye [4]. Additionally, in 75% of patients visual acuity did not differ by more than two Snellen lines between eyes. Among those patients with 6/6 vision, over 90% complained of visual deficits with over 80% reporting blurry vision on presentation. This raises the suggestion that perhaps it is metamorphopsia, rather than true loss of visual acuity responsible for the visual complaints in these patients.

In our study, the most common symptoms on presentation in descending order were floaters, poor vision, photopsia, and nyctalopia. This fits in well with previous data in the literature, namely Shah et al. reporting blurry vision as the most common, followed by floaters, nyctalopia, and dyschromatopsia [4]. Priem et al. reported similar figures [36].

In terms of clinical signs, our study revealed optic disc swelling to be the most common, followed by CMO and optic disc pallor. These findings are in close agreement with another European study which reported the presence of macular oedema in up to a third of eyes, depending on whether optical coherence tomography or fluorescein angiography criteria were used. Retinal vasculitis was noted in almost half of eyes, which may contribute to optic disc oedema and atrophy [38, 39].

The authors wish to highlight that presenting features of BSCR are often non-specific and can masquerade as other conditions. Particularly, patients may complain of poor vision in the initial stages of the disease in the absence of objective evidence of visual decline. This often leads to delayed and even missed diagnoses until late stages of the disease.

Limitations in disease recognition include the exclusion of BSRC based on the absence of birdshot lesions in the early phase of the disease, or the misattribution of the fundal lesions to another of the ‘white dot syndromes’. While the presence of these lesions has been classically described in BSRC, they are not disease-defining in the early phase [40]. In an attempt to ease diagnostic challenges, it has been suggested that HLA-A*29 positivity and stromal choroiditis evident on indocyanine green angiography (ICGA) are sufficient for the diagnosis of BSRC [41, 42].

Functional tests such as electroretinography (ERG), perimetry and colour vision form an indispensable part of the diagnostic arsenal in BSCR. It is important to note that these tests may show abnormal changes even in the presence of normal BCVA [36, 43–49]. In particular, the role of ERG as an objective test in the monitoring of disease activity has been previously described [45], with one study even reporting on ERG improvement preceding clinical recovery in a subset of patients [50]. It is interesting to note that over half of reporting ophthalmologists in this study indicated ‘unknown’ for at least one of these parameters, despite the fact that functional disturbances as a reflection of disease progression (particularly on ERG) may be observed even in the presence of normal VA. In the context of BSCR diagnostic challenges, the authors wish to draw attention to this and encourage wider use of functional tests as part of the work-up for this disease.

Currently, there is a crucial gap in the literature with regards to optimum treatment duration and regime for BSCR. It is often the case that this disease is managed using locally authored guidelines, based on clinician experience and published posterior uveitis guidelines. Given the high and prolonged steroid doses usually required to induce remission, early initiation of steroid-sparing immunomodulatory therapy is widely accepted for the preservation of visual integrity and reduction of structural ocular damage [51]. Various immunosuppressant regimes have been described across study groups. The FOCUS initiative conferred the use of mycophenolate monotherapy or in combination with cyclosporine as level 2B/3 evidence [52–54]. The use of infliximab was described as level 2B/3B [55, 56] or level 4 evidence [57, 58]. Few studies have reported on the modest benefit of cyclosporine in BSRC [59, 60], while one arm of the SITE Cohort Study outlined its role in non-infectious posterior uveitis [61]. Another arm of the SITE study described the efficacy of methotrexate as an addition to corticosteroid therapy, but again did not specify the individual uveitis diagnoses [62]. The authors found 58.4% of patients to achieve complete steroid-sparing effect (prednisone ≤10 mg/d), while 66% achieved control of inflammation at one year [62]. Another large retrospective case series evaluating the efficacy of methotrexate in chronic non-infectious uveitis found it to be effective, with 56% of patients successfully achieving the steroid-sparing effect [63]. Though, BSRC comprised a very small proportion of the study’s patient population. Another small retrospective case series study reported good efficacy with tacrolimus, with 80% of patients achieving steroid-sparing effect (prednisone ≤10 mg/d) at one year [64].

The VISUAL-1 and VISUAL-2 clinical trials highlighted the role of adalimumab therapy in improving health-related and vision-related quality of life in patients with non-infectious uveitis [65–67]. With BSRC patients comprising a significant sub-set of the study population, these trials showed adalimumab to be associated with a lower risk of uveitic flare or visual impairment compared to placebo.

Other treatment options include topical, periocular, and intravitreal steroids, often used in cases of acute or recurrent CMO, with systemic steroids used as initial therapy as a bridge to immunomodulatory agents [59, 68–70]. In this study, most patients received steroids in combination with IMT at initial diagnosis. At twelve months, however, the proportion of patients receiving steroid-sparing agents alone increased, while those using topical steroids significantly declined. It is conceivable that in some patients, topical steroids were commenced by general ophthalmologists prior to referral to uveitis specialists, for the management of CMO and vitritis. The fact that a higher proportion of patients than would be expected are initially on topical and periocular therapy raises the question regarding initial ambiguity of diagnosis. In the absence of standardised guidelines for BSCR, this study aims to raise awareness of the high number of patients inappropriately initiated on topical steroid therapy, and highlight the need for validated treatment recommendations.

In conclusion, BSRC is a rare progressive posterior uveitis with the potential for significant visual morbidity. This study confirms the pre-existing body of knowledge related to BSCR, and aims to educate ophthalmologists on the difficulty in timely diagnosis of BSCR, the need for comprehensive functional testing and the importance of steroid-sparing treatment. To the authors’ knowledge, this is the first prospective surveillance study of this disease in a high prevalence region such as the UK.

### Study limitations

Since BOSU relies on voluntary reporting by consultant ophthalmologists, sources of error include forgetting to report a case, failing to recall a patient’s details, the inability to retrieve patient notes, losing the yellow card, or the inconvenience of using the postal system. Other systematic barriers to participation include lack of complete understanding of study inclusion criteria, or the dates of the study period, and hesitation to report adverse treatment outcomes [71, 72]. A particular challenge we faced was ophthalmologists not returning the initial or follow-up questionnaire after indicating a new case on the yellow card. We addressed this by sending at least two additional reminders with moderate success.

Another limitation of this study was the lack of case ascertainment obtained through an external source. One way of performing this would be to contact a number of tertiary uveitis referral centres at random, and ascertain whether they kept a database of BSRC patients at their institution, for comparison to data in returned yellow cards and our own questionnaires [71]. Another approach would be to randomly select a number of reporting consultants and ask if they received the yellow reporting cards in the post, and whether or not they had any cases to report [73]. These external validation techniques would assist in mitigating the bias of under-reporting, however are labour intensive and logistically challenging.

Although major referral centres like Manchester and London reported a relatively high number of cases in this study, others were not as well engaged. There were a high number of duplicate reports, which was not unexpected given many patients attend several centres before diagnosis, resulting in duplicate reporting.

In addition to the high number of excluded cases, and likelihood of delayed and missed diagnoses in real-life settings, it is possible that the true incidence of BSCR is higher than stated in this study. However, within the established reporting system of the BOSU, as well as the additional year of data acquisition employed by the authors, we believe this study represents the most accurate data to date of this rare disease.

It is important to note that the effect of BSCR on visual outcomes is complex; visual decline may be central, peripheral, related to colour, or due to loss of contrast sensitivity, and as such cannot be solely represented by BCVA. Additionally, vision may be confounded by various other pathologies such as cataract. Due to the small number of participants in this study, we were unable to account for these factors when reporting on visual acuity. In future, a statistical adjustment for these biases would be useful.

Of note, there is currently no standardised grading of disease severity in BSCR. In future, a severity scale could be devised in an attempt to standardise reporting of cases, with contributions from both functional assessments and structural findings, although this is outside the confines of this study. In doing so it should be accepted that BCVA would be of low contributor to the score, as it is possible to have severe disease in the presence of normal acuity.

Another limitation of this study was the lack of sufficient information on visual fields, ERG findings and colour vision analysis, allowing only for a small sub-analysis as related to presenting visual acuity. Additionally, our questionnaire only asked clinicians to delineate whether these were normal, abnormal or unknown. At least half of all eligible cases reported “unknown” for at least one of these parameters, despite their necessary role in the diagnosis of BSCR. It must be emphasised that markers of disease activity and progression include not only patient-reported symptoms and slit lamp findings, but also those of electroretinography, colour vision testing and perimetry. The authors aim to spread awareness of the need for rigorous functional testing as an adjunct to disease monitoring, and thus treatment decisions.

### Summary

#### What was known before

- Birdshot Retinochoroiditis (BSRC) is a chronic, sight-threatening posterior uveitis with a predisposition to females of Northern European origin. Treatment usually consists of long-term systemic immunosuppressive therapy. This disease, and the treatment thereof, impacts quality of life. Although described as rare, some countries have higher prevalence; typically Northern Europe, which is likely over-represented due to the figures being based on data from tertiary referral centres. To date, there is no accurate country-wide incidence data from a high-prevalence region.

#### What this study adds

- We do not know if the incidence of BSRC is increasing, and an accurate incidence would provide a benchmark which would prove helpful for clinicians and researchers. To the authors' knowledge, this is the first prospective country-wide surveillance study to report on the incidence and presenting clinical findings of BSRC, in a high-prevalence region such as the UK. In this 2-year study, the incidence of BSRC in the UK was 0.035 cases per 100,000 person-years. We did not find a significant difference in the geographic distribution of BSRC across the UK, probably owing to the small number of cases. We were also unable to map deprivation scores due to insufficient postcode information. Although no specific treatment guidelines exist for BSRC, therapy usually consists of a combination of steroids and immunomodulatory agents. For the treatment of acute flares, local or systemic steroids are used as first-line therapy.

## Supplementary information

Supplementary material

## Data Availability

The datasets generated during and/or analysed during the current study are available from the corresponding author on reasonable request.
