# Supplementary material for: The incidence, presenting clinical findings and treatment patterns of Birdshot Retinochoroiditis in a high-prevalence region: findings from Northern Ireland, England and Wales
Source: Eye (Lond). 2023 Feb 10;37(13):2817–25. doi: 10.1038/s41433-023-02425-y (PMC10482920; doi:10.1038/s41433-023-02425-y)
Supplement: Supplementary file 1 — Supplementary material [file 41433_2023_2425_MOESM1_ESM.docx]

Supplementary material 1

*Details required on the questionnaires sent to reporting ophthalmologists:*

1. Patient demographics
2. Duration of symptoms
3. Date of diagnosis
4. Best corrected visual acuity
5. Presenting eye symptoms
6. Clinical signs at diagnosis
7. Examination findings including colour vision, visual field testing and electrodiagnostic testing
8. Past ophthalmic history
9. Past medical history of psoriasis
10. Family history of BSRC
11. Treatment started for BSRC
12. Treatment started for other inflammatory eye disease including previous and current usage

Supplementary material 2: Initial questionnaire

**BOSU Birdshot Retinochoroidopathy Surveillance Study: Initial Questionnaire**

**Patient details:**

Hospital number _____________ Ethnicity: Caucasian ⬜

Month and Year of Birth _____/_______ Afro-Caribbean ⬜

Asian ⬜

Gender: Male ⬜ Female ⬜ Far-Eastern ⬜

Country of Birth _____________ Mixed (any) ⬜

First half of Post code _____________

**Referral:**

Duration of symptoms______________________

Date of diagnosis of Birdshot ____/____/_______

Was the patient transferred from another unit?

**❑** No ❑ Yes (*Please Specify)___________________________________*

*(NB: This is to check for duplicate reporting. We will not contact other clinicians.)*

Was the patient transferred to another unit?

**❑** No ❑ Yes (*Please Specify)_____________________________________*

*(NB: This is to check for duplicate reporting. We will not contact other clinicians.)*

**Presenting Eye Symptoms:** Right eye Left eye

Photopsia ⬜No ⬜Yes ⬜No ⬜ Yes

Floaters ⬜No ⬜Yes ⬜No ⬜ Yes

Nyctalopia ⬜No ⬜Yes ⬜No ⬜ Yes

Poor/reduced vision ⬜No ⬜Yes ⬜No ⬜ Yes

**Past Ophthalmic History:** Right Eye Left eye

Previous rhegmatogenous retinal detachment: No ⬜ Yes ⬜

Other: _____________________ No ⬜ Yes ⬜

**Past Medical History of Psoriasis?** No ⬜ Yes ⬜

**Family History of Birdshot**? No ⬜ Yes ⬜

**HLA-A29 status:**  ⬜ +ve ⬜ -ve

**Examination findings at diagnosis:**

Best Corrected Visual Acuity: Right Eye _______Left eye _______

(⬜ Snellen ⬜ logMAR ⬜ EDTRS)

Colour Vision: ⬜ Normal ⬜ Abnormal ⬜ Unknown

Visual Field: ⬜ Normal ⬜ Abnormal ⬜ Unknown

Electrodiagnostic Tests: ⬜ Normal ⬜ Abnormal ⬜ Unknown

**Presence at diagnosis of:** Right Eye Left eye

Cystoid macular oedema: ⬜No ⬜Yes ⬜No ⬜ Yes

Optic disc swelling: ⬜No ⬜Yes ⬜No ⬜ Yes

Optic disc pallor: ⬜No ⬜Yes ⬜No ⬜ Yes

Choroidal neovascular membrane⬜No ⬜Yes ⬜No ⬜ Yes

OTHER CAUSES OF REDUCED VISION:

(Give details) ⬜No ⬜ Yes_______ ⬜No ⬜ Yes_______

**Please give details of any immunosuppressive medication for:**

Other inflammatory eye disease:

⬜No ⬜Yes_____________ ⬜No ⬜ Yes_____________

Currently using: Previously used:

Topical

⬜No ⬜Yes_____________ ⬜No ⬜ Yes_____________

Periocular

⬜No ⬜Yes_____________ ⬜No ⬜ Yes_____________

Intravitreal

⬜No ⬜Yes_____________ ⬜No ⬜ Yes_____________

Systemic

⬜No ⬜Yes_____________ ⬜No ⬜ Yes_____________

**Treatment started for BSRC:**

Topical Steroid

⬜No ⬜ Yes ____________________________________

Periocular Steroid

⬜No ⬜ Yes____________________________________

Intravitreal steroid

⬜No ⬜ Yes____________________________________

Systemic Steroid

⬜No ⬜ Yes If YES, starting dose _____mg/day

Other Systemic Immunosuppressant: No ⬜ Yes ⬜

Name of medication 1._______________Date started____/____/________

Name of medication 2._______________Date started____/____/________

Further Comments ____________________________________________________

Doctor Completing Questionnaire ________________________________________

Ophthalmic Unit_______________________________________________________

Supplementary material 3: Follow-up questionnaire

**BOSU Birdshot Retinochoroidopathy Surveillance Study:**

**Follow-up Questionnaire**

**Patient details:**

Hospital number __________________

DOB _____/_________

Gender: Male ⬜ Female ⬜

First half of current postcode _____

**Referral:**

Date of diagnosis of Birdshot ________________

Is the working diagnosis still Birdshot Retinochoroidopathy? ⬜Yes ⬜No

(If NO then please state revised diagnosis and no further answers are required__________________________________________________________).

Thank you for your time in completing this questionnaire.

Was the patient transferred to another unit?

**❑** No ❑ Yes (*Please Specify)_________________________________________*

*(NB: This is to check for duplicate reporting. We will not contact other clinicians.)*

**Current treatment for BSRC:**

Topical Steroid ⬜No ⬜ Yes

Periocular Steroid ⬜No ⬜ Yes

Intravitreal steroid ⬜No ⬜ Yes

Systemic Steroid ⬜No ⬜ Yes If YES, current dose _____mg/day

Other Systemic Immunosuppressant: No ⬜ Yes ⬜

Name of medication 1._______________Date started____/____/________

Name of medication 2._______________Date started____/____/________

**Current immunosuppressive medication for:**

Other inflammatory disease:

⬜No ⬜Yes_____________

**Eye Symptoms at 12 months:** Right eye Left eye

Photopsia ⬜No ⬜Yes ⬜No ⬜ Yes

Floaters ⬜No ⬜Yes ⬜No ⬜ Yes

Nyctalopia ⬜No ⬜Yes ⬜No ⬜ Yes

Poor/reduced vision ⬜No ⬜Yes ⬜No ⬜ Yes

**Examination findings at 12 months:**

Visual Acuity: ⬜ Snellen ⬜ logMAR ⬜ EDTRS

Best corrected Right Eye ___________ Left eye ___________

Colour Vision: ⬜ Normal ⬜ Abnormal ⬜ Unknow

Visual Field: ⬜ Normal ⬜ Abnormal ⬜ Unknown

Electrodiagnostic Tests: ⬜ Normal ⬜ Abnormal ⬜ Unknown

**Presence at 12 months of:** Right Eye Left eye

Cystoid macular oedema: ⬜No ⬜Yes ⬜No ⬜ Yes

Optic disc swelling: ⬜No ⬜Yes ⬜No ⬜ Yes

Optic disc pallor: ⬜No ⬜Yes ⬜No ⬜ Yes

Choroidal neovascular membrane⬜No ⬜Yes ⬜No ⬜ Yes

Is this patient eligible for CVI registration? ⬜No ⬜ Yes

Further Comments __________________________________________________________________

___________________________________________________________________

**THANK YOU FOR TAKING THE TIME TO COMPLETE THIS QUESTIONNAIRE**

Supplementary material 4: Findings from functional tests at diagnosis

|  | **Mean BCVA >LogMAR 0.2** | **Mean BCVA ≦LogMAR 0.2** | **Total patients (n)** |
| --- | --- | --- | --- |
| **Normal visual fields**  **(both eyes)** | 1/9 (11%) | 8/9 (89%) | 9 |
| **Abnormal visual fields**  **(≧1 eye)** | 6/8 (75%) | 2/8 (25%) | 8 |
| **Total patients** | 7 | 10 | 17 |

|  | **Mean BCVA**  **>LogMAR 0.2** | **Mean BCVA ≦LogMAR 0.2** | **Total patients (n)** |
| --- | --- | --- | --- |
| **Normal ERG (both eyes)** | 0/2 (0%) | 2/2 (100%) | 2 |
| **Abnormal ERG**  **(≧1 eye)** | 5/9 (56%) | 4/9 (44%) | 9 |
| **Total patients** | 5 | 6 | 11 |

|  | **Mean BCVA >LogMAR 0.2** | **Mean BCVA ≦LogMAR 0.2** | **Total patients(n)** |
| --- | --- | --- | --- |
| **Normal colour vision**  **(both eyes)** | 4/16 (25%) | 12/16 (75%) | 16 |
| **Abnormal colour vision**  **(≧1 eye)** | 2/2 (100%) | 0/2 (0%) | 2 |
| **Total patients** | 6 | 12 | 18 |
